# Management of Hepatitis B Virus in Allogeneic Hematopoietic Stem Cell Transplantation

**DOI:** 10.3389/fimmu.2020.610500

**Published:** 2021-02-04

**Authors:** Yibo Wu, He Huang, Yi Luo

**Affiliations:** ^1^Bone Marrow Transplantation Center, the First Affiliated Hospital, Zhejiang University School of Medicine, Hangzhou, China; ^2^Institute of Hematology, Zhejiang University, Hangzhou, China; ^3^Zhejiang Laboratory for Systems & Precision Medicine, Zhejiang Province Engineering Laboratory for Stem Cell and Immunity Therapy, Hangzhou, China; ^4^Zhejiang Laboratory for Systems & Precision Medicine, Zhejiang University Medical Center, Hangzhou, China

**Keywords:** hepatitis B virus, hematopoietic stem cell transplantation, HBV resolved infection, HBV reactivation, HBV-related hepatitis, stem cell donor

## Abstract

The high morbidity of HBV reactivation following allogeneic hematopoietic stem cell transplantation (allo-HSCT) is partially due to the intense immunologic potency of complex therapeutic regimens, the use of antithymocyte globulin and calcineurin inhibitors to prevent graft versus-host disease (GVHD), prolonged immune reconstitution, and hematological malignancies infected with hepatitis B virus (HBV). Immunosuppression results in the reactivation of HBV replication from covalently closed circular DNA (cccDNA) residing in hepatocytes. However, the role of viral mutations during HBV reactivation needs to be validated. All individuals scheduled to receive allo-HSCT or wish to donate stem cells should be screened for hepatitis B surface antigen (HBsAg), antibodies to hepatitis B core (anti-HBc), and HBV-DNA. HBsAg-positive recipients of allo-HSCT have a high risk of HBV reactivation; thus, they should receive prophylactic antiviral therapy. The high barrier to resistance nucleos(t)-ide analogs (NAs) seems to be superior to the low barrier agents. Resolved-HBV recipients have a lower risk of HBV reactivation than HBsAg-positive recipients. Although prophylactic antiviral therapy remains controversial, regular monitoring of alanine transaminase (ALT) and HBV-DNA combined with preemptive antiviral treatment may be an optimized strategy. However, optimal antiviral therapy duration and time intervals for monitoring remain to be established. Accepting stem cells from HBsAg-positive donors is associated with a risk of developing HBV-related hepatitis. The overall intervention strategy, including donors and recipients, may decrease the risk of HBV-related hepatitis following HSCT from HBsAg positive stem cells. In this review, we summarize the issues of HBV in allo-HSCT, including HBV reactivation mechanism, HBsAg-positive recipients, HBV-resolved infection recipients, and donor-related factors, and discuss their significance.

## Introduction

Globally, an estimated 257 million people live with chronic HBV infection (1). The HBV carrier rate is high (6.2%) in the African and Western Pacific regions (2). In China, the prevalence rate of hepatitis B surface antigen (HBsAg) was estimated at 5–6%, and 4.38% of people 15–29 years of age are carriers (3, 4). Researchers are aware of HBV reactivation (HBVr) complications in patients receiving chemotherapy, monoclonal antibody (especially anti-CD20 antibody) treatment, and other intensive immunosuppressive therapies. Since covalently closed circular DNA (cccDNA) persists in hepatocytes and other tissues, HBsAg-positive patients and historically HBV infected patients are at a risk of HBVr during immunosuppressive therapy (5–7). The strength of HBVr is determined by the degrees of immune control and virus immune activity *in vivo*. In addition, because of the intense immunologic potency of the complex therapeutic regimens, and the use of rituximab and high-dose glucocorticoids, which usually leads to cytopenia, the incidence of HBVr due to immunosuppression is much higher in hematological malignancies than in other diseases (8, 9).

Guidelines have been recommended for patients with HBV infection undergoing immunosuppressive and cytotoxic therapy (8, 10–14). Hematopoietic stem cell transplantation (HSCT) technology has developed rapidly and is expected to become the mainstay treatment for patients with hematologic malignancies. Myeloablative conditioning regimens, antithymocyte globulin and calcineurin inhibitor treatment to prevent graft-versus-host disease (GVHD), high-dose glucocorticoids for GVHD therapy, prolonged immune reconstitution, evolving therapeutic treatments (e.g. ruxolitinib, rituximab, ibrutinib, and monoclonal antibodies) for chronic GVHD therapy, and the risk of donor HBV sources lead to a heighted risk of HBVr complication in hematological patients who accept allo-HSCT. Allo-HSCT is an independent risk factor for HBVr in patients with hematologic malignancies (15). However, knowledge regarding HBVr in allo-HSCT is not comprehensive and there are no standard guidelines for managing HBVr during allo-HSCT. Due to the high probability of HBV infection in hematological patients living in HBV epidemic area and the high frequency of HBVr during HSCT, it is necessary to review the developments made by studies on HBVr in allo-HSCT in recent decades. To comprehensively understand HBVr in allo-HSCT and help physicians deal with HBVr in allo-HSCT, here, we have summarized and reviewed the key issues in this domain.

## Definition of HBV Reactivation

**Figure 1:**
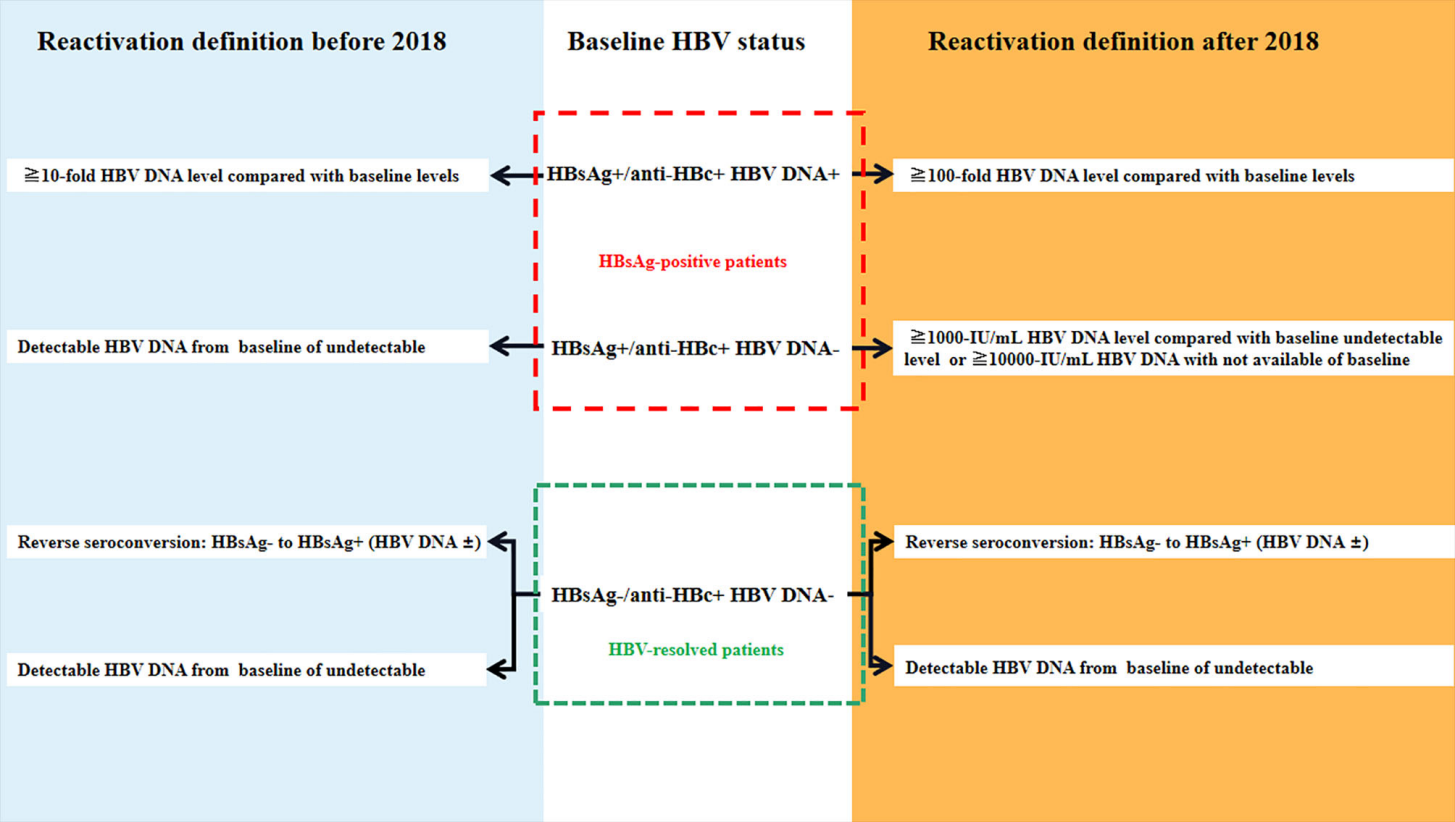
The definition of hepatitis B virus (HBV) reactivation [adapted from (13)].

Previously there were no standard criteria for HBVr. For HBsAg-positive patients, HBVr was defined as a) 10-fold elevation of circulating HBV DNA compared with baseline levels before HSCT and b) detectable circulating HBV DNA in patients whose serum HBV DNA was undetectable before HSCT. In HBV-resolved patients, HBVr was defined as a) a positive result for the HBsAg test in a patient who previously tested negative (called reverse seroconversion, RS) and b) detectable circulating HBV DNA in patients with undetectable serum HBV DNA before HSCT (6, 13, 16, 17) (**Figure 1**). In 2018, the American Association for the Study of Liver Diseases (AASLD) recommended stricter criteria for HBVr (10). For HBsAg-positive patients, (1) ≥2 log (100-fold) increase in HBV DNA compared to the baseline, (2) HBV DNA ≥ 3 log (1,000) IU/ml in a patient with previously undetectable levels, (given that HBV DNA levels fluctuate) or (3) HBV DNA ≥ 4 log (10,000) IU/ml, if the baseline level is not available. For patients who are anti-HBc-positive and HBsAg-negative, the criteria are: (1) detection of HBV DNA, or (2) reappearance of HBsAg (**Figure 1**). Increase in alanine transaminase (ALT) levels to ≥3 times the baseline level and >100 U/L was deemed a hepatitis flare and the definition of HBV related to hepatitis was hepatitis flare plus HBVr.

## HBV Reactivation Mechanism

**Figure 2:**
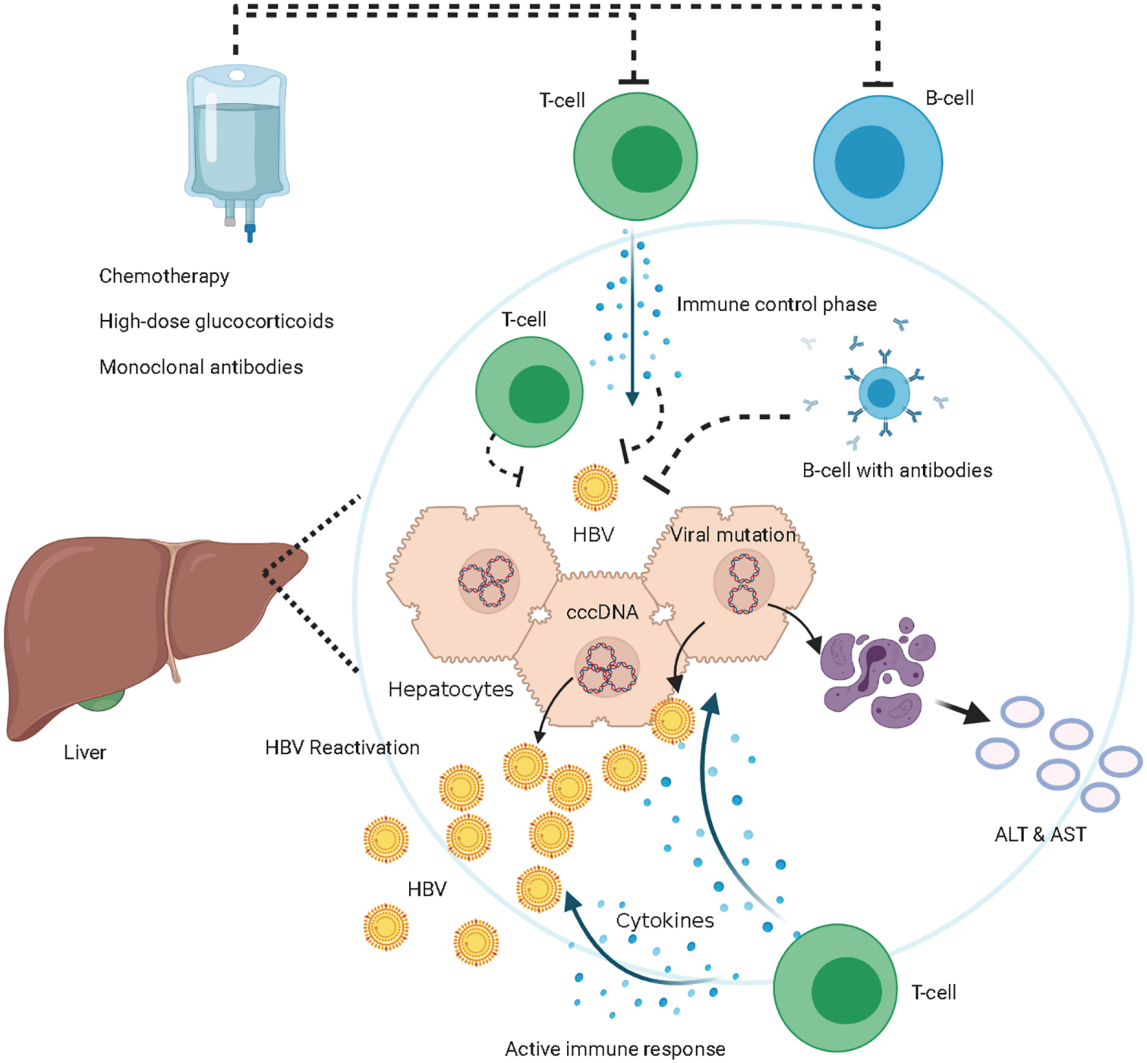
The mechanism of hepatitis B virus (HBV) reactivation. Immune control phase, HBV-specific T-cell suppress viral replication by both cytopathic effects and non-cytopathic cytokine pathways, B cells produce antibodies against HBV and inhibit the spread of HBV infection among hepatocytes, HBV cccDNA persistent in hepatocytes. Immune suppressive phase, T-cells and B-cells are inhibited or eliminated by the immunosuppressive treatments, HBV DNA restarts replication due to treatment-induced loss of immune control. Maybe HBV mutations lead to immune-escape from HBV-specific T-cell, HBV DNA restarts replication. HBV reactivation occurs when HBV DNA amplify actively *in vivo*. Active immune phase, immune system reconstitution and T-cells attack HBV-DNA and HBV infected hepatocytes. The destructed hepatocytes release ALT and aspartate aminotransferase (AST).

HBV enters the body and eventually enters hepatocytes through the key liver-specific receptor, sodium-taurocholate co-transporter (18). The nucleocapsid is inserted into the nucleus of hepatocytes and the DNA is converted into cccDNA (19). HBV cccDNA is stable and persistent in hepatocytes, which is the reservoir of HBVr despite serum clearance of HBV (20, 21) (**Figure 2**). The host’s immune response to HBV infection undergoes an inactive immune tolerance state, active state, and conversion to the immune control phase (22, 23). HBV-specific T-cell responses suppress viral replication by both cytopathic effects and non-cytopathic cytokine pathways (24, 25). B cells produce antibodies against HBV and inhibit the spread of HBV infection to other hepatocytes (**Figure 2**). The first report of HBVr was made in 1975 by Wands in a patient with lymphoproliferative disease undergoing chemotherapy (26). It has been reported that HBV DNA restarts replication due to treatment-induced loss of immune control and immunosuppression (27–29).

In addition, HBV mutations in the major hydrophilic region of the S domain have been found in HBVr after HSCT in recent years (30–35). However, there is still no evidence that immune-escape mutations occurred prior to reactivation, or that they are responsible for assisting in the viral reactivation process (30). The role of viral mutations and immune escape during HBVr needs to be validated. There was no phenomenon of HBV-specific CD8 T-cell exhaustion during HBVr reported. Hepatitis occurs after immune system reconstitution and destruction of HBV infected hepatocytes (36) (**Figure 2**). HBV immune response activities with amplification of HBV DNA cannot be detected in time since they precede ALT alterations and clinical symptoms. HBV-induced hepatocellular damage is considered to be the result of a complex interplay among HBV, hepatocytes, and immune cells of the host; thus, several HBVr patients may have little or no liver dysfunction, while others may have hepatitis flares, interruption of immunosuppressive drugs, hepatic failure, and even death. The serological alteration of HBV and the HBV DNA load *in vivo* cannot effectively reflect the clinical influence of HBVr after allo-HSCT (37). Recent studies have found a favorable prognosis for HBVr in allo-HSCT recipients (38–42). The RS of HBV-resolved patients facilitates hepatitis B surface antigen seroclearance following antiviral treatment (38, 39). However, previous studies had different HBVr definitions and heterogeneous patient characteristics. Therefore, it is necessary to reevaluate the clinical influence of HBVr in a homogeneous group following consistent HBVr criteria, such as the incidence of HBV-related hepatitis, liver-related mortality, non-relapse mortality, and interruption or reduction of primary immunosuppressants.

## HBsAg-Positive Recipients

HBsAg-positive recipients have been widely recognized as high-risk (>10%) for HBVr following allo-HSCT. A study from Mary Hospital of the University of Hong Kong in 2002 indicated that the historical control without prophylactic antiviral treatment had an incidence of 45% HBVr after allo-HSCT (43). A multicenter retrospective study from the Italian Group for Blood and Marrow Transplantation showed that two-year incidence of HBVr after allo-HSCT in HBsAg-positive recipients was up to 81% without prophylactic antiviral treatment (44). Because of the high risk of HBVr, there were no HBVr results reported in HBsAg-positive allo-HSCT recipients without prophylactic antiviral treatment, due to thoughtful ethical considerations. Many perspective studies have demonstrated the validity of prophylactic lamivudine (LAM) 100 mg daily in decreasing the risk of HBVr in HBsAg-positive patients receiving chemotherapy (45–47). Similarly, prophylactic LAM 100 mg daily decreased the risk of HBVr to 5% (1/20) in HBsAg-positive recipients following allo-HSCT (43). Furthermore, other studies have shown the effectiveness of prophylactic entecavir (ETV) 0.5 mg daily in minimizing HBVr in HBsAg-positive recipients following allo-HSCT (48–50). In addition, the high barrier to resistance NAs (ETV, tenofovir disoproxil fumarate [TDF], or tenofovir alafenamide [TAF]) seem to be superior to the low barrier to resistance antiviral drugs (LAM, Telbivudine [LdT], and Adefovir [ADV]). A retrospective study from China in 2016 indicated that the ETV 0.5 mg daily group had a much lower incidence of HBVr than the LAM 100 mg daily group (2.1%[2/97] vs. 23.5%[28/119],p<0.001) for HBsAg-positive recipients following allo-HSCT (51). However, data comparing the high barrier to resistance of NAs (ETV, TDF, or TAF) with low barrier agents (LAM, LdT, ADV) was limited. Physicians performing HSCT may be concerned whether the antiviral drug would influence the engraftment of neutrophils or platelets during allo-HSCT. A retrospective study in Brazil indicated that LAM/ETV/TAF treatment had no influence on neutrophil or platelet engraftment in allo-HSCT, which needs to be confirmed in large-sample size studies (52). Based on these studies, nearly all guidelines for the prevention of HBVr associated with immunosuppressive therapy had consensus regarding screening for HBsAg and HBcAb before accepting allo-HSCT treatments (11, 13, 53, 54), and prophylactic antiviral treatment was recommended to decrease the risk of HBVr for HBsAg-positive recipients. However, there is no explicit view on screening for HBV DNA and hepatitis B surface antibody (HBsAb) before accepting allo-HSCT therapy. Additionally, the monitoring interval of serological HBV, the duration of prophylactic antiviral treatment, and choice of NAs remain unclear. One suggestion has been that referred monitoring should continue for 6 months after cessation of immunosuppressive therapy, with 2-month intervals potentially being appropriate (55). A recent publication suggested that serological HBV should be obtained at baseline and evaluated every 6 months during antiviral therapy. Additionally, it should also be checked at least monthly for the first 3 months after the cessation of antiviral therapy and every 3 months thereafter (56). Although there is no consensus on duration of antiviral treatment, the majority of recommendations for prophylactic antiviral treatment duration vary from 6 months to 12 months after discontinuation of immunosuppressive therapy (10, 11, 13, 54).

## HBV-Resolved Recipients

HBV-resolved patients were defined as HBsAg-negative but positive for antibodies against hepatitis B core antigen (57). While HBV was not actively amplifying *in vivo*, the persistent cccDNA in hepatocytes could be amplified again when the patient’s immune system is suppressed. Previous prospective studies have shown that HBV-resolved hematological patients who accept immunosuppressive treatment/chemotherapy have a risk of HBVr, with the incidence of HBVr varying from 8.3% to 41.5% (7, 58–62). However, the risk of HBVr in HBV-resolved patients who accept allo-HSCT is not well known. A multicenter retrospective study from Italy illustrated that 6/50 (12%) of HBV-resolved patients underwent HBVr after allo-HSCT without prophylactic antiviral treatment at a median of 12 (7–32) months after transplantation; the 5-year cumulative incidence of HBVr was 22% (63). A retrospective study from Chiba University found that 4/35 (11%) HBV-resolved patients developed HBVr (64) without prophylactic antiviral treatment. Another retrospective study from San Martino University Hospital indicated that 14/137 (10%) patients had HBVr within a median of 19 months (range 9–77 months) after HSCT (40) without prophylactic antiviral treatment. We found that 13/300 (4.3%) HBV-resolved patients developed HBVr at a median of 588 days (range 455–1,294 days) after HSCT without prophylactic antiviral treatment (39). A recent retrospective study with a similar sample size from the National Taiwan University Hospital reported that 21/485 (4.72%) HBV-resolved patients presented HBVr at 16 months (range 8–50 months) after HSCT with no antiviral prophylaxis (42). A higher risk of HBVr was reported by a retrospective study at Hamanomachi Hospital, Japan: 18/69 (26.1%) HBV-resolved patients developed HBVr after allo-HSCT at a median of 440 days (75–1,829) without prophylactic antiviral treatment (65). The only prospective research in the domain of HBV-resolved patients undergoing allo-HSCT was conducted by the University of Hong Kong. The two-year cumulative incidence of HBVr was 40.8% (13/62) without prophylactic antiviral treatment, occurring at a median of 44 (8–100) weeks post-transplantation (41). Based on these studies, HBsAg-negative, anti-HBc-positive patients who underwent allo-HSCT also had a risk of HBV reactivation. However, it remains unclear whether prophylactic antiviral therapy can benefit HBV-resolved patients following allo-HSCT treatment as much as it benefits HBsAg-positive patients. A retrospective study from the University of Genoa evaluated 7 years’ worth of single-center data on HBV-resolved patients who received allo-HSCT; none of the 50 HBV-resolved patients experienced HBVr while on prophylactic LAM treatment (66). However, another study indicated that although the majority of HBV-resolved recipients accepted antiviral treatment, the rates of HBVr in the HSCT group at one and seven years were 2.5% and 57.9%, respectively (38).

There are many controversial issues in HBV-resolved patients receiving allo-HSCT. The risk stratification of HBsAg-negative/HBcAb-positive HSCT recipients and recommendations on antiviral treatment are inconsistent from different specialized associations (**Table 1**). The Asian Pacific Association for the Study of the Liver recommended in 2015 that HBsAg-negative/HBcAb-positive patients with undetectable serum HBV DNA should be followed carefully by means of ALT and HBV DNA testing, then treating with NAs therapy upon confirmation of HBVr. Most European and American specialized associations provide aggressive views on prophylactic antiviral therapy in HBV-resolved patients. The experts in the domain of immunosuppressive treatment-related HBVr recommend initiating prophylactic treatment or monitoring HBV DNA levels for HBsAg-negative/HBcAb-positive patients undergoing intermediate-risk immunosuppression (6, 58). However, whether HBV-resolved patients receiving allo-HSCT belong to the high-risk or intermediate-risk group of HBVr is controversial. Previous studies reported a much lower incidence of HBVr in the HBsAg-negative/HBcAb-positive group than HBsAg-positive patients who underwent allo-HSCT. Large-sample prospective studies are needed to robustly investigate the incidence of HBVr in HBV-resolved patients undergoing allo-HSCT according to the 2018 AASLD definition of HBVr. Moreover, the effectiveness of prophylactic antiviral therapy in minimizing the risk of HBVr for HBV-resolved HSCT recipients is not well known. There are no studies comparing high barrier NAs (ETV, TDF, or TAF) with low barrier agents (LAM, LdT, and ADV) in HBsAg-negative/HBcAb-positive allo-HSCT recipients. The monitoring interval of serological HBV and duration of prophylactic antiviral treatment is also unclear (**Table 1**).

**Table 1:** Recommendations for hepatitis B surface antigen (HBsAg)-negative/HBcAb-positive patients undergoing allo-hematopoietic stem cell transplantation (HSCT) from different specialized associations.

Association	Risk stratification	Screen recommendation	Recommendation	Duration	Reference
American Society of Clinical Oncology Provisional Clinical Opinion 2015	High risk	Screen for HBsAg and HBcAb, followed by a sensitive HBV DNA test if positive	Prophylactic antiviral therapy or monitored closely and start antiviral therapy if HBVr occurs	Continued up to 12 months after cessation of therapy	(13)
American Gastroenterological Association Institute 2015	Not reported	Screen for HBsAg and HBcAb, followed by a sensitive HBV DNA test if positive	Antiviral prophylaxis	Continue for at least 6 months after discontinuation of immunosuppressive therapy (12 months for B cell–depleting agents).	(11)
Asian Pacific Association for the Study of the Liver 2015	Not reported	Screen for HBsAg and HBcAb prior to treatment, tested for HBV DNA if HBcAb-positive	Patients with detectable HBV DNA should antiviral treatment, patients with undetectable HBV DNA should be followed carefully by ALT and HBV DNA testing, and be treated with NA therapy upon confirmation of HBVr	Not reported	(67)
European Society of Clinical Microbiology and Infectious Diseases 2017	Not reported	Screen for HBsAg, HBcAb and HBsAb, followed by a sensitive HBV DNA test if positive of HBsAg/HBcAb	Prophylaxis with LAM, independent of the presence of HBV DNA	At least 18 months	(53)
European Association for the Study of the Liver 2017	High risk	Screen for HBsAg, HBsAb and HBcAb	Antiviral prophylaxis	Continue for at least 18 months after stopping immunosuppression	(54)
The Indian National Association for Study of the Liver 2018	Not reported	Screen for HBsAg and HBcAb, tested for HBV DNA if HBcAb-positive	Monitored with HBsAg, ALT and HBV DNA every 3 months during therapy and up to 6 months, pre-emptive antiviral therapy on detection of HBsAg or HBV DNA positivity	Continued for at least 18 months after discontinuation of HSCT	(14)
The American Association for the Study of Liver Diseases 2018	Lower risk of HBVr than HBsAg-positive patients, and depending on their clinical situation	Screen for HBsAg and HBcAb	Antiviral prophylaxis	Continued for at least 12 months after completion of immunosuppressive therapy	(10)
American Society of Clinical Oncology 2020	High risk	Screen for HBsAg, anti-HBc, and HBsAb	Antiviral prophylaxis Or careful monitoring and antiviral therapy at the earliest sign of HBVr	Continue for minimum 12 months after anticancer therapy completion	(56)

## Underlying Reasons Why Controversies Exist in Prophylactic Antiviral Therapy of HBV-Resolved Allo-HSCT Recipients

The protective role of HBsAb has been found in hematological patients receiving chemotherapy. A systematic review described the protective role of the HBsAb, with a lower HBV reactivation rate in HBsAb(+) patients compared with HBsAb(-) patients with hematologic disease (7.1% versus 21.8%; P < 0.001) (9). In a group of HBsAg(-)/HBcAb(+) patients with lymphoma, patients without HBsAb before rituximab-based chemotherapy had a higher incidence of HBV reactivation than those with HBsAb (68.3% vs. 34.4%; P = 0.01) (61). It was reported that exceeding the threshold HBsAb titer of 100 IU/ml was associated with a 0% rate of HBVr (68, 69). However, few studies have investigated the role of HBsAb in HBcAb-positive patients who undergo HSCT. The role of HBsAb during HBVr in HSCT is unclear. We stratified 665 HBsAg-negative patients according to HBcAb/HBsAb presence into four groups; the HBcAb(+)HBsAb(-) group had the highest risk of HBVr among the patient groups (15.7%; P < 0.001). The cumulative HBV reactivation rates were 5.3% in the HBcAb(+)HBsAb(+) group, 0% in the HBcAb(-)HBsAb(-) group, and 2.1% in the HBcAb(-)HBsAb(+) group, with no significant difference among these groups. HBsAb in HSCT recipients conferred a protective effect against HBVr (39). A recent retrospective study from Turkey also reported the protective role of HBsAb in HBVr during allo-HSCT (70). Twenty two HBV-resolved patients showed different two-year cumulative incidence of HBVr (20% vs 75%) between groups of HBcAb(+)HBsAb(+) and HBcAb(+)HBsAb(-) (70). Certainly, these need to be verified in larger prospective studies. Nearly all studies on HBVr in HBsAg-negative/HBcAb-positive allo-HSCT recipients reported that HBVr was a late phase complication (**Table 2**). Additionally, nearly all studies recommended the administration of 6–12 months of antiviral prophylaxis. It is necessary to consider the value of early antiviral prophylactic treatment in preventing late phase complications of HBVr.

**Table 2:** Time of hepatitis B virus (HBV) reactivation in hepatitis B surface antigen (HBsAg)-negative/HBcAb-positive recipients after hematopoietic stem cell transplantation (HSCT).

Publish year	Number of HBVr	Type of study	Type of transplantation	Regimen	Probability of HBVr (cumulative rate)	HBV reactivation time after allo-HSCT	Reference
2011	6/50 (12.0%)	retrospective	45 MSD/2 HRD/3 MUD	35MAC/15RIC	13% at 1 year, 22% at 5 years	12 (range 7–32) months	(63)
2014	14/137 (10.2%)	retrospective	76 MSD/20 HRD/24 MUD/17 Others	63MAC/74RIC	6.3% at 2 years, 9.6% at 5 years	19 (range 9–77) months	(40)
2014	4/35 (11.4%)	retrospective	Not reported	Not reported	Not reported	19 months	(64)
2015	3/11 (27.3%)	prospective	3 Allo/7 Auto/1 Auto plus Allo	Not reported	Not reported	8, 9, 10 months	(15)
2016	14/52 (26.9%)	retrospective	Not reported	30MAC/22RIC	10.8% at 1 year, 43.9% at 5 years	15 (range 3–68) months	(71)
2017	7/107 (6.5%)	retrospective	Auto	Not reported	3.5% at 1 year, 5% at 2 years	16 (range 7–47) months	(72)
2017	13/62 (20.9%)	prospective	Not reported	41MAC/21RIC	17.7% at 1 year, 40.8% at 2 years	44 (range 8–100) weeks	(41)
2019	50/385 (12.9%)	retrospective	Not reported	Not reported	2.5% at 1 year, 57.9% at 7 years	19.9 (range 2.4–75.6) months	(38)
2019	18/69 (26.1%)	retrospective	Not reported	Not reported	11.2% at 1 year, 43.0% at 5 years	440 (range 75–1,829) days	(65)
2019	21/445 (4.72%)	retrospective	Not reported	196MAC/249RIC	2.2% at 1 year, 10.5% at 5 years	16 (range 8–50) months	(42)
2020	13/300 (4.3%)	retrospective	77 MSD/149 HRD/74 MUD	300MAC/0RIC	Not reported	645 (range 455–1,957) days	(39)

MSD, matched sibling donor; HRD, haploidentical related donor; MUD, matched unrelated donor; MAC, myeloablative conditioning; RIC, reduced intensity conditioning; Allo, allogeneic.

However, long-term antiviral treatment may cause resistance. The cumulative incidence of HBV resistance of anti-HBV drugs with a low resistance barrier (LAM, LdT, and ADV) is prevalent and growing over time in patients with chronic hepatitis B (54). LAM resistance occurs in up to 20% of patients after just one year of use (8). One study reported that an HBsAg-negative patient who underwent allo-HSCT using stem cells from an HBsAg-positive donor eventually acquired HBV infection due to a YYMD mutation as a result of long-term prophylactic treatment with LAM (73). One report suggested that HBV reemerges with T127P, F170FL, and S204R mutations with prophylactic LAM treatment, causing HBVr after HSCT (34). Furthermore, recent studies found favorable prognosis of HBVr in HBsAg-negative/HBcAb-positive HSCT recipients (38–42). HBVr in these groups can be controlled, and most HBVr patients acquire serologic clearance of HBsAg with regularly HBVr monitoring and preemptive antiviral treatment (38, 39). After antiviral initiation, HBV-resolved patients with reactivation showed a one year cumulative HBsAg clearance of 68.3% (38). No case of serologic clearance in an HBV-RS patient who recovered from HBVr converted to active HBsAg carriers has been reported. The liver-related mortality of HBVr was nearly zero. In a large prospective study monitoring HBV DNA monthly in HBV-resolved B cell lymphoma patients, no hepatitis due to HBVr was observed in patients who received antiviral treatment when HBV DNA levels were between 11 and 432 IU/ml (7). Another prospective study enrolled 83 HBsAg-negative/HBcAb-positive hematologic patients receiving anti-CD20 therapy. These patients were monitored every 4 weeks without antiviral therapy and every 2 weeks once HBV DNA was detectable. All patients with HBV DNA had RS or two-fold increase in upper limit of normal ALT received antiviral therapy. After therapy, ALT was normalized and HBV DNA returned to undetectable levels. There were no cases of clinical hepatitis, liver failure, or death (74). These data indicate that regular monitoring and preemptive antiviral therapy are effective methods for preventing HBVr-related hepatitis in HBV-resolved patients following immunosuppressive therapy.

## Donor Related Factors

It was first confirmed in 1995 that HBV can be transmitted *via* stem cells from HBsAg-positive donors to recipients during HSCT (75). A group at Queen Mary Hospital in Hong Kong pioneered the application of stem cells from HBsAg-positive donors in allo-HSCT (16, 76, 77). The incidence of HBV-related hepatitis in recipients who receive HBsAg-positive donor stem cells is in the range of 48% to 55.5% (16, 76). Therefore, serological HBsAg positivity seems to be a contraindication for HSCT donors, due to the fear of HBV-related hepatitis. Selecting a suitable donor for a HSCT recipient from a pool of potential HBsAg-positive donors is an unresolved problem. The donor’s predicted favorable factors for HSCT outcomes may conflict with the status of serologic HBsAg positivity. There are no standard guidelines for managing patients who receive stem cells from HBsAg-positive donors. The Fifth European Conference on Infections in Leukemia indicated that both the donor and recipient undergo antiviral treatment and that HBsAg-negative recipients are vaccinated to prevent HBV transmission (78). These measures lowered the incidence of HBV-related hepatitis to 6.9% of recipients who receive stem cells from HBsAg-positive donors, whereas the historical control group was 48% (16). We established a protocol for the management of HBsAg-positive donors comprising of antiviral treatment to lower circulating HBV DNA levels in HBsAg-positive donors, induction of passive immunity in HbsAg-negative recipients using hepatitis B immune globulin, and prophylactic antiviral treatment of HBsAg-positive recipients. The five-year cumulative incidence of HBV-related hepatitis was comparable in patients who received stem cells from HBsAg-positive donors and matched control recipients who received stem cells from HbsAg-negative donors (8.5% [95% CI, −0.9% to 17.9%] vs. 7.9% [95% CI, −0.9% to 16.7%]; P = 0.939) (79). Thus, the overall intervention strategy for accepting HBsAg-positive donors may expand the application of allo-HSCT in HBV-endemic areas by allowing for the inclusion of HBsAg-positive donors. All of the strategies for dealing with accepting HBsAg-positive donors need to be tested in a well-designed prospective study.

## Adoptive Immune Transfer

It was reported that donor HBsAb decreased the risk of HBVr in HSCT recipients (40). Univariate and multivariate analyses of HBVr risk factors confirmed the protective role of an HBV-immune/exposed donor (HR adjusted = 0.12, 95% CI 0.02–0.96; P = 0.045) (40). A recent large retrospective study indicated that the cumulative incidence of HBV-RS at 5 years was 16.3% and 8.4% for patients with or without donor anti-HBs, respectively. Multivariate analysis revealed that the independent risk factor for HBV-RS was allo-HSCT from donors lacking anti-HBs compared with other donors with anti-HBs antibodies (HR= 0.294; 95% CI, 0.13–0.85; P = 0.0117) (42). However, we stratified 565 HBsAg-negative donors according to HBcAb and HBsAb status into four groups. The cumulative HBVr rates at 5 years in the four groups were 5.3% for HBcAb(-)HBsAb(-), 5.1% in HBcAb(-)HBsAb(+), 3.8% in HBcAb (+)HBsAb(-), and 1.6% in HBcAb(+)HBsAb(+) (P=0.794). We did not find a protective role for HBV-immune/exposed donors in HSCT recipients (39). In addition, there were few reports of HBsAg clearance in HBsAg-positive patients after allo-HSCT. There were several case reports demonstrating that an HBV-immune/exposed donor with HBsAb can lead to serologic clearance of HBsAg in HBsAg-positive recipients (80, 81). The factors influencing HBsAg clearance in HBsAg-positive patients following allo-HSCT are unclear. Additionally, there was no strong evidence that adoptive immune transfer plays a protective role in HBVr during allo-HSCT.

## HBV Vaccine Issues

The Francophone Society of Bone Marrow Transplantation and Cellular Therapy recommended HBV vaccine for HBV-resolved recipients to prevent HBVr after allo-HSCT (82). A retrospective study from Hokkaido University Hospital enrolled 21 patients with HBV-resolved infection (83). They received a standard three-dose regimen of hepatitis B vaccine after discontinuation of immunosuppressants. The first vaccine was administered at a median of 15 months (range, 6–79 months) after transplantation. Nine of them tested positive for HBsAb. None of the 21 patients in the vaccine group developed HBVr, which indicated that HBV vaccination of HSCT recipients was a promising method for preventing HBVr. However, the following multicenter prospective clinical research of hepatitis B vaccine to prevent HBVr after allo-HSCT failed to find the protective role of hepatitis B vaccine in minimizing the risk of HBVr in HBV-resolved recipients (31). Only 37% (10/27) of patients had HBsAb with three doses of hepatitis B vaccine 12 months after HSCT, and the 2-year cumulative incidence of HBVr was 27.3%. Encouragingly, a recent preliminary study showed excellent results with an anti-HBs seroconversion rate of 82% in HBsAg-negative pediatric and young adult recipients after HSCT at the median of 10.4 (range 3.0–22.4) months after the third vaccination (84). Another prospective study enrolled 86 adults that accepted a low dose of the HBV vaccine (10 mg/dose) at 6, 7, 8, 12 months after allo-HSCT. The proportion of recipients achieving anti-HBs antibody titers 100 mUI/ml was 64.6% (95% CI, 53% to 75%; n = 51/79) at 6 months after vaccine initiation and 56.8% (95% CI, 39.5% to 72.9%; n = 21/37) at 24 months after vaccine initiation (85). This study suggested a better efficacy of higher HBV vaccine antigen doses. However, the effectiveness of these vaccines in preventing HBVr remains to be evaluated.

## OBI (Occult Hepatitis B Infection)

OBI was defined as the presence of replication-competent HBV DNA in the liver and/or HBV DNA in the blood of people who test negative for HBsAg. These patients can be classified as seropositive OBI (HBsAb-positive or HBcAb-positive) and seronegative OBI (HBsAb-negative and HBcAb-negative) (86). A study found that 19/124 (15.3%) HBsAg-negative donors were detected to have OBI by using the PCR method, for which 14/19 (73.7%) OBI donors were HBsAb-positive (77). Thus, transmission of stem cells from OBI blood donors is a risk factor for HBV-related hepatitis. Patients with OBI have a risk of HBVr when they receive cancer chemotherapy or other immunosuppressive therapies (86). The risk is high (>10%) in OBI patients receiving anti-CD20 containing regimens and myeloablative regimens for HSCT (41, 61, 62, 87). Considering the probability of OBI, both HSCT recipients and donors should be screened for HBV DNA before HSCT.

## New Therapeutic Treatments

Several new treatment strategies have emerged for patients after allo-HSCT. These include CAR-T therapy and blinatumomab therapy for relapse, rituximab, ruxolitinib, ibrutinib, and other monoclonal antibodies for chronic GVHD treatment. However, as these strategies target B cells and/or T cells, they may also cause HBVr. Indeed, rituximab is known for resulting in HBVr (59). HBV reactivation has also been reported after CAR-T therapy in patients with current or past HBV infection (88–91). Cases of HBVr have been associated with ibrutinib treatment for hematological malignancies but not for patients after allo-HSCT (92–94). Therefore, physicians should carefully monitor the ALT and HBV DNA in chronic HBV infection or resolved HBV infection patients when applying new therapeutic treatments after allo-HSCT.

## Future Directions

Several unresolved issues remain regarding HBVr during allo-HSCT, which require more work in future studies. The new DNA sequence of HBVr in the process of HBVr needs to be found to redefine the role of virus mutation itself in the mechanism of HBVr. The frequency of monitoring and the duration of NAs prophylactic administration in preventing HBVr during allo-HSCT remains unclear and needs to be established urgently. Many consensuses still recommend LAM as the first-line option. The data regarding the comparison of high barrier (ETV, TDF, or TAF) NAs with low barrier agents (LAM, LdT, ADV) in the prophylaxis of HBVr in HSCT is limited. The frequency of developing drug resistance during long-term antiviral treatment, especially for low barrier agents, in HSCT is unclear. These data may change the first-line recommendation of prophylactic antiviral treatment for HBVr in the future. Prophylactic antiviral therapy for HBV-resolved patients is controversial and large-sample prospective studies are needed to investigate the incidence of HBVr in these patients following allo-HSCT. The protective role of HBsAb in HBVr during allo-HSCT and the adoptive immune effect of donors for decreasing HBVr are awaiting further exploration. Regularly monitoring of ALT and HBV DNA and preemptive antiviral treatment in HBV-resolved patients need to be verified. We believe that HBsAb-positive patients may be the most likely stratified group of resolved HBV infection patients who need not accept prophylactic antiviral therapy for preventing HBVr. The dosages and schedule of vaccines in preventing HBVr in HBV-resolved recipients remains to be evaluated in prospective trials. The clinical influence of HBVr during allo-HSCT requires further investigation, such as the interruption of immunosuppressants, severe hepatitis, liver-related mortality, and non-relapse mortality. We portray an agenda for future further research in HBVr during allo-HSCT (**Table 3**).

**Table 3:** Agenda for further research.

Group	Further research	Purpose
HBsAg-positive recipients	The frequency of monitoring and the duration of NAs prophylactic administration	Establish antiviral therapy duration and monitored time intervals
	Comparison effectiveness and resistance of high barrier drugs and low barrier agents in the prophylaxis of HBVr	Promote to first-line recommendation of prophylactic antiviral treatment with high barrier agents
	HBsAg clearance in HBsAg-positive patients after allo-HSCT accepting stem cells from HBsAb-positive donors	To verify the adoptive immune role and the influence factors of HBsAg Seroclearance
	New therapeutic treatments (e.g. ruxolitinib, ibrutinib, monoclonal antibodies)	To investigate the latent risk of HBVr during treatments
HBsAg-negative/HBcAb-positive recipients	Prospective studies to reflect the real cumulative rate of HBVr without prophylactic treatment	To investigate the incidence of HBVr in HBV-resolved patients undergoing allo-HSCT
	Prospective studies to investigate the protective role of HBsAb (both recipients and donors)	To explore the protective role of HBsAb in HBVr
	The frequency of monitoring and the duration of NAs prophylactic administration	Establish antiviral therapy duration and monitored time intervals
	HBV vaccine dosage and schedule for minimizing the risk of HBVr	Explore and establish valid method of preventing HBVr with vaccine method
	New therapeutic treatments (e.g. ruxolitinib, rituximab, ibrutinib, monoclonal antibody)	To investigate the latent risk of HBVr during treatments
HBsAg-positive donors	Prospective clinical trials to verify the strategy for preventing HBV related hepatitis from accepting HBsAg positive donors	To establish the effective strategy for accepting stem cells from HBsAg positive donors
All HBVr recipients	Investigate the viral mutation with gene sequencing method	To verify the role of virus itself in process of HBVr during allo-HSCT
	Investigate the incidence of HBV-related hepatitis, liver-related mortality, non-relapse mortality, and interruption of immunosuppressants	To reevaluate the clinical influence of HBVr after allo-HSCT

## Conclusions

Considering the risk of HBVr, all individuals who plan to receive allo-HSCT or donate stem cells should be screened for HBsAg, HBcAb, and HBV DNA. HBsAg-positive recipients of allo-HSCT have a high risk of HBVr. They should accept prophylactic antiviral therapy to decrease the risk of HBVr. The high barrier NAs (ETV, TDF, or TAF) seem to be superior to the low barrier agents (LAM, LdT, ADV). Resolved HBV infection recipients also have a risk of HBVr, but the risk is lower than that of HBsAg-positive recipients. There are controversies in prophylactic antiviral therapy for resolved HBV infection recipients to prevent HBVr. The optimal antiviral therapy duration and monitored time intervals in both HBsAg-positive and HBsAg-negative/HBcAb-positive recipients remain to be established. There is little evidence to suggest that adoptive donor immunity plays an important role in the prevention of HBVr after allo-HSCT. Accepting stem cells from HBsAg-positive donors is associated with a risk of viral infection, and thus may develop HBV-related hepatitis. The overall intervention strategy, including donors and recipients, can decrease the risk of HBV-related hepatitis following HSCT from HBsAg-positive stem cells. It will increase the treatment options for patients in need of allo-HSCT in HBV-endemic areas by allowing the inclusion of HBsAg-positive individuals as donors.

## Author Contributions

YW, writing of the original draft. HH, funding acquisition, project administration, and validation. YL, funding acquisition, project administration, review, and validation. All authors contributed to the article and approved the submitted version.

## Funding

This work was supported by grants from The National Key Research and Development Program of China (2018YFA0107804) and the National Natural Science Foundation of China (81970158).

## Conflict of Interest

The authors declare that the research was conducted in the absence of any commercial or financial relationships that could be construed as a potential conflict of interest.

## References

[1] World Health Organization Global hepatitis report (2017). Available at: https://www.who.int/hepatitis/publications/global-hepatitis-report2017/en/ (Accessed September 25, 2020).

[2] Yuen MF Chen DS Dusheiko GM Janssen HLA Lau DTY Locarnini SA Hepatitis B virus infection. Nat Rev Dis Primers (2018) 4(1):18035–. 10.1038/nrdp.2018.35 29877316

[3] Liu J Liang W Jing W Liu M Countdown to 2030: eliminating hepatitis B disease, China. Bull World Health Organ (2019) 97(3):230–8. 10.2471/BLT.18.219469 PMC6453311 30992636

[4] Cui F Shen L Li L Wang H Wang F Bi S Prevention of Chronic Hepatitis B after 3 Decades of Escalating Vaccination Policy, China. Emerg Infect Dis (2017) 23(5):765–72. 10.3201/eid2305.161477 PMC5403029 28418296

[5] Roche B Samuel D The difficulties of managing severe hepatitis B virus reactivation. Liver Int (2011) 31(Supplement s1):104–10. 10.1111/j.1478-3231.2010.02396.x 21205146

[6] Gonzalez SA Perrillo RP Hepatitis B Virus Reactivation in the Setting of Cancer Chemotherapy and Other Immunosuppressive Drug Therapy. Clin Infect Dis (2016) 62(suppl 4):S306–S13. 10.1093/cid/ciw043 PMC4889897 27190320

[7] Kusumoto S Tanaka Y Suzuki R Watanabe T Nakata M Takasaki H Monitoring of Hepatitis B Virus (HBV) DNA and Risk of HBV Reactivation in B-Cell Lymphoma: A Prospective Observational Study. Clin Infect Dis (2015) 61(5):719–29. 10.1093/cid/civ344 25935551

[8] Pattullo V Prevention of Hepatitis B reactivation in the setting of immunosuppression. Clin Mol Hepatol (2016) 22(2):219–37. 10.3350/cmh.2016.0024 PMC4946398 27291888

[9] Cholongitas E Haidich A-B Apostolidou-Kiouti F Chalevas P Papatheodoridis GV Hepatitis B virus reactivation in HBsAg-negative, anti-HBc-positive patients receiving immunosuppressive therapy: a systematic review. Ann Gastroenterol (2018) 31(4):480–90. 10.20524/aog.2018.0266 PMC6033767 29991894

[10] Terrault NA Lok AS McMahon BJ Chang KM Hwang JP Jonas MM Update on prevention, diagnosis, and treatment of chronic hepatitis B: AASLD 2018 hepatitis B guidance. Hepatology (2018) 67(4):1560–99. 10.1002/hep.29800 PMC5975958 29405329

[11] Reddy KR Beavers KL Hammond SP Lim JK Falck-Ytter YT American Gastroenterological Association Institute Guideline on the Prevention and Treatment of Hepatitis B Virus Reactivation During Immunosuppressive Drug Therapy. Gastroenterology (2015) 148(1):215–9. 10.1053/j.gastro.2014.10.039 25447850

[12] Chinese Society of Hematology Committee of Malignant Lymphoma Chinese Anti-cancer Association Chinese Society of Hepatology Consensus on the management of lymphoma with HBV infection. Chin J Hematol (2013) 34(11):988–93. 10.3760/cma.j.issn.0253-2727.2013.11.019

[13] Hwang JP Somerfield MR Alston-Johnson DE Cryer DR Feld JJ Kramer BS Hepatitis B virus screening for patients with cancer before therapy: American Society of Clinical Oncology provisional clinical opinion update. J Clin Oncol (2015) 33(19):2212–20. 10.1200/JCO.2015.61.3745 PMC4477791 25964247

[14] Arora A Anand AC Kumar A Singh SP Aggarwal R Dhiman K INASL Guidelines on Management of Hepatitis B Virus Infection in Patients receiving Chemotherapy, Biologicals, Immunosupressants, or Corticosteroids. J Clin Exp Hepatol (2018) 8(4):403–31. 10.1016/j.jceh.2018.06.010 PMC6286881 30568345

[15] Pompili M Basso M Hohaus S Bosco G Nosotti L D’Andrea M Prospective study of hepatitis B virus reactivation in patients with hematological malignancies. Ann Hepatol (2015) 14(2):168–74. 10.1016/S1665-2681(19)30778-1 25671825

[16] Hui C Lie A Au W Ma S Leung YH Zhang H Effectiveness of prophylactic anti-HBV therapy in allogeneic hematopoietic stem cell transplantation with HBsAg positive donors. Am J Transplant (2005) 5(6):1437–45. 10.1111/j.1600-6143.2005.00887.x 15888052

[17] Perrillo RP Gish R Falck-Ytter YT American Gastroenterological Association Institute technical review on prevention and treatment of hepatitis B virus reactivation during immunosuppressive drug therapy. Gastroenterology (2015) 148(1):221–44.e3. 10.1053/j.gastro.2014.10.038 25447852

[18] Yan H Zhong G Xu G He W Jing Z Gao Z Sodium taurocholate cotransporting polypeptide is a functional receptor for human hepatitis B and D virus. Elife (2012) 1:e00049. 10.7554/eLife.00049 23150796 PMC3485615

[19] Hu J Protzer U Siddiqui A Revisiting Hepatitis B Virus: Challenges of Curative Therapies. J Virol (2019) 93(20):e01032–19. 10.1128/JVI.01032-19 PMC6798116 31375584

[20] Revill PA Chisari FV Block JM Dandri M Gehring AJ Guo H A global scientific strategy to cure hepatitis B. Lancet Gastroenterol Hepatol (2019) 4(7):545–58. 10.1016/S2468-1253(19)30119-0 PMC6732795 30981686

[21] Rehermann B Ferrari C Pasquinelli C Chisari FV The hepatitis B virus persists for decades after patients’ recovery from acute viral hepatitis despite active maintenance of a cytotoxic T-lymphocyte response. Nat Med (1996) 2(10):1104–8. 10.1038/nm1096-1104 8837608

[22] World Health Organization Guidelines for the Prevention, Care and Treatment of Persons with Chronic Hepatitis B Infection. Geneva: World Health Organization (2015). 26225396

[23] Gentile G Antonelli G HBV Reactivation in Patients Undergoing Hematopoietic Stem Cell Transplantation: A Narrative Review. Viruses (2019) 11(11):1049. 10.3390/v11111049 PMC6893755 31717647

[24] Moriyama T Guilhot S Klopchin K Moss B Pinkert C Palmiter R Immunobiology and pathogenesis of hepatocellular injury in hepatitis B virus transgenic mice. Science (1990) 248(4953):361–4. 10.1126/science.1691527 1691527

[25] Kakimi K Lane T Wieland S Asensio V Campbell I Chisari FV Blocking chemokine responsive to gamma-2/interferon (IFN)-gamma inducible protein and monokine induced by IFN-gamma activity in vivo reduces the pathogenetic but not the antiviral potential of hepatitis B virus-specific cytotoxic T lymphocytes. J Exp Med (2001) 194(12):1755–66. 10.1084/jem.194.12.1755 PMC2193580 11748277

[26] Wands J Subacute and chronic active hepatitis after withdrawal of chemotherapy. Lancet (1975) 2(7942):979. 10.1016/s0140-6736(75)90391-8 53460

[27] Galbraith RM Williams R Eddleston ALWF Zuckerman AJ Bagshawe KD Fulminant hepatic failure in leukaemia and choriocarcinoma related to withdrawal of cytotoxic drug therapy. Lancet (1975) 306(7934):528–30. 10.1016/s0140-6736(75)90897-1 51345

[28] Perrillo RP Acute flares in chronic hepatitis B: the natural and unnatural history of an immunologically mediated liver disease. Gastroenterology (2001) 120(4):1009–22. 10.1053/gast.2001.22461 11231956

[29] Tur-Kaspa R Shaul Y Moore DD Burk RD Okret S Poellinger L The glucocorticoid receptor recognizes a specific nucleotide sequence in hepatitis B virus DNA causing increased activity of the HBV enhancer. Virology (1988) 167(2):630–3. 10.1016/0042-6822(88)90127-4 3201757

[30] Lazarevic I Banko A Miljanovic D Cupic M Immune-escape hepatitis B virus mutations associated with viral reactivation upon immunosuppression. Viruses (2019) 11(9):778. 10.3390/v11090778 PMC6784188 31450544

[31] Nishikawa K Kimura K Kanda Y Sugiyama M Kakihana K Doki N A prospective trial of vaccine to prevent hepatitis B virus reactivation after hematopoietic stem cell transplantation. Bone Marrow Transplant (2020) 55(7):1388–98. 10.1038/s41409-020-0833-5 PMC7329632 32071416

[32] Cerva C Colagrossi L Maffongelli G Salpini R Di Carlo D Malagnino V Persistent risk of HBV reactivation despite extensive lamivudine prophylaxis in haematopoietic stem cell transplant recipients who are anti-HBc-positive or HBV-negative recipients with an anti-HBc-positive donor. Clin Microbiol Infect (2016) 22:946.e1–.e8. 10.1016/j.cmi.2016.07.021 27475741

[33] Chen PM Yao NS Wu CM Yang MH Lin YC Hsiao LT Detection of reactivation and genetic mutations of the hepatitis B virus in patients with chronic hepatitis B infections receiving hematopoietic stem cell transplantation. Transplantation (2002) 74(2):182–8. 10.1097/00007890-200207270-00007 12151729

[34] Cerva C Maffongelli G Svicher V Salpini R Colagrossi L Battisti A Hepatitis B reactivation characterized by HBsAg negativity and anti-HbsAg antibodies persistence in haematopoietic stem cell transplanted patient after lamivudine withdrawal. BMC Infect Dis (2017) 17(1):566. 10.1186/s12879-017-2672-6 28806922 PMC5557326

[35] Inuzuka T Ueda Y Arasawa S Takeda H Matsumoto T Osaki Y Expansion of viral variants associated with immune escape and impaired virion secretion in patients with HBV reactivation after resolved infection. Sci Rep (2018) 8(1):18070. 10.1038/s41598-018-36093-w 30584239 PMC6305382

[36] Brugger SA Oesterreicher C Hofmann H Kalhs P Greinix HT Hepatitis B virus clearance by transplantation of bone marrow from hepatitis B immunised donor. Lancet (British Ed) (9057) 1997) 349:996–7. 10.1016/s0140-6736(05)62893-0 9100630

[37] Visram A Feld JJ Defining and grading HBV reactivation. Clin Liver Dis (2015) 5(2):35–8. 10.1002/cld.426 PMC6490456 31040945

[38] Lee HL Jang JW Han JW Lee SW Bae SH Choi JY Early Hepatitis B Surface Antigen Seroclearance Following Antiviral Treatment in Patients with Reactivation of Resolved Hepatitis B. Digest Dis ences (2019) 64:2992–3000. 10.1007/s10620-019-05614-6 30982209

[39] Zhang A Wu Y Tan Y Shi J Zhao Y Hu Y Determining Whether Prophylactic Antiviral Treatment Is Necessary in HBsAg-Negative/HBcAb-Positive Patients Receiving Allogeneic Hematopoietic Stem Cell Transplantation. Biol Blood Marrow Transplant (2020) 26(5):956–64. 10.1016/j.bbmt.2020.01.006 31962163

[40] Mikulska M Nicolini L Signori A Rivoli G Del Bono V Raiola AM Hepatitis B reactivation in HBsAg-negative/HBcAb-positive allogeneic haematopoietic stem cell transplant recipients: risk factors and outcome. Clin Microbiol Infect (2014) 20(10):694–701. 10.1111/1469-0691.12611 24575948

[41] Seto W-K Chan TS-Y Hwang Y-Y Wong DK-H Fung J Liu KS-H Hepatitis B reactivation in occult viral carriers undergoing hematopoietic stem cell transplantation: A prospective study. Hepatology (2017) 65(5):1451–61. 10.1002/hep.29022 28027590

[42] Liu JH Liao XW Chen CH Yao M Li CC Lin CT Adoptive donor immunity protects against resolved hepatitis B virus reactivation after allogeneic haematopoietic stem cell transplantation in the world’s largest retrospective cohort study. Br J Haematol (2019) 186(1):72–85. 10.1111/bjh.15884 30919947

[43] Lau GK He ML Fong DY Bartholomeusz A Wy A AK L Preemptive use of lamivudine reduces hepatitis B exacerbation after allogeneic hematopoietic cell transplantation. Hepatology (2002) 36(3):702–9. 10.1053/jhep.2002.35068 12198664

[44] Locasciulli A Bruno B Alessandrino E Meloni G Arcese W Bandini G Italian Cooperative Group for Blood and Marrow Transplantation. Hepatitis reactivation and liver failure in haemopoietic stem cell transplants for hepatitis B virus (HBV)/hepatitis C virus (HCV) positive recipients: a retrospective study by the Italian group for blood and marrow transplantation. Bone Marrow Transplant (2003) 31(4):295–300. 10.1038/sj.bmt.1703826 12621466

[45] Hwang JP Fisch MJ Zhang H Kallen MA Routbort MJ Lal LS Low rates of hepatitis B virus screening at the onset of chemotherapy. J Oncol Pract (2012) 8(4):e32–e9. 10.1200/JOP.2011.000450 PMC3396827 23180996

[46] Lau GK Yiu HH Fong DY Cheng H-C Au W-Y Lai LS Early is superior to deferred preemptive lamivudine therapy for hepatitis B patients undergoing chemotherapy. Gastroenterology (2003) 125(6):1742–9. 10.1053/j.gastro.2003.09.026 14724827

[47] Hsu C Hsiung CA Su IJ Hwang WS Wang MC Lin SF A revisit of prophylactic lamivudine for chemotherapy-associated hepatitis B reactivation in non-Hodgkin’s lymphoma: a randomized trial. Hepatology (2008) 47(3):844–53. 10.1002/hep.22106 18302293

[48] Liao Y-P Jiang J-L Zou W-Y Xu D-R Li J Prophylactic antiviral therapy in allogeneic hematopoietic stem cell transplantation in hepatitis B virus patients. World J Gastroenterol (2015) 21(14):4284–92. 10.3748/wjg.v21.i14.4284 PMC4394091 25892880

[49] Aoki J Kimura K Kakihana K Ohashi K Sakamaki H Efficacy and tolerability of Entecavir for hepatitis B virus infection after hematopoietic stem cell transplantation. SpringerPlus (2014) 3:450. 10.1186/2193-1801-3-450 25184113 PMC4149683

[50] Tsuji M Ota H Nishida A Ishiwata K Yamamoto H Yamamoto G Entecavir is safe and effective as prophylaxis for reactivation of hepatitis b virus in allogeneic stem cell transplant recipients with chronic or resolved viral hepatitis b infection. Blood (2012) 120(21):4144. 10.1182/blood.V120.21.4144.4144

[51] Shang J Wang H Sun J Fan Z Huang F Zhang Y A comparison of lamivudine vs entecavir for prophylaxis of hepatitis B virus reactivation in allogeneic hematopoietic stem cell transplantation recipients: a single-institutional experience. Bone Marrow Transplant (2016) 51(4):581–6. 10.1038/bmt.2015.328 26752138

[52] Buzo BF Ramos JF Rossetti RAM Salles N Mendrone-Júnior A Rocha V Hepatitis B virus among hematopoietic stem cell transplant recipients: Antiviral impact in seroconversion, engraftment, and mortality in a Latin American center. Transplant Infect Dis (2020) 22(2):e13243. 10.1111/tid.13243 31901206

[53] Sarmati L Andreoni M Antonelli G Arcese W Bruno R Coppola N Recommendations for screening, monitoring, prevention, prophylaxis and therapy of Hepatitis B virus reactivation in patients with haematological malignancies and patients who underwent haematological stem cell transplantation - a position paper. Clin Microbiol Infect (2017) 23(12):935–40. 10.1016/j.cmi.2017.06.023 28668466

[54] European Association for the Study of the Liver EASL 2017 Clinical Practice Guidelines on the management of hepatitis B virus infection. J Hepatol (2017) 67(2):370–98. 10.1016/j.jhep.2017.03.021 28427875

[55] Bisceglie AMD Lok AS Martin P Terrault N Perrillo RP Hoofnagle JH Recent US Food and Drug Administration warnings on hepatitis B reactivation with immune-suppressing and anticancer drugs: just the tip of the iceberg? Hepatology (2015) 61(2):703–11. 10.1002/hep.27609 PMC5497492 25412906

[56] Hwang JP Feld JJ Hammond SP Wang SH Alston-Johnson DE Cryer DR Hepatitis B Virus Screening and Management for Patients With Cancer Prior to Therapy: ASCO Provisional Clinical Opinion Update. J Clin Oncol (2020) 38(31):3698–715. 10.1200/JCO.20.01757 PMC11828660 32716741

[57] Kusumoto S Arcaini L Hong X Jin J Kim WS Kwong YL Risk of HBV reactivation in patients with B-cell lymphomas receiving obinutuzumab or rituximab immunochemotherapy. Blood (2019) 133(2):137–46. 10.1182/blood-2018-04-848044 PMC6337873 30341058

[58] Law MF Ho R Cheung CK Tam LH Ma K So KC Prevention and management of hepatitis B virus reactivation in patients with hematological malignancies treated with anticancer therapy. World J Gastroenterol (2016) 22(28):6484–500. 10.3748/wjg.v22.i28.6484 PMC4968128 27605883

[59] Yeo W Chan TC Leung NW Lam WY Mo FK Chu MT Hepatitis B virus reactivation in lymphoma patients with prior resolved hepatitis B undergoing anticancer therapy with or without rituximab. J Clin Oncol (2009) 27(4):605–11. 10.1200/JCO.2008.18.0182 19075267

[60] Huang Y-H Hsiao L-T Hong Y-C Chiou T-J Yu Y-B Gau J-P Randomized controlled trial of entecavir prophylaxis for rituximab-associated hepatitis B virus reactivation in patients with lymphoma and resolved hepatitis B. J Clin Oncol (2013) 31(22):2765–72. 10.1200/JCO.2012.48.5938 23775967

[61] Seto W-K Chan T Hwang Y-Y Wong D Fung J Liu K Hepatitis B reactivation in patients with previous hepatitis B virus exposure undergoing rituximab-containing chemotherapy for lymphoma: a prospective study. J Clin Oncol (2014) 32(33):3736–43. 10.1200/JCO.2014.56.7081 25287829

[62] Hsu C Tsou HH Lin SJ Wang MC Yao M Hwang WL Chemotherapy-induced hepatitis B reactivation in lymphoma patients with resolved HBV infection: a prospective study. Hepatology (2014) 59(6):2092–100. 10.1002/hep.26718 24002804

[63] Viganò M Vener C Lampertico P Annaloro C Pichoud C Zoulim F Risk of hepatitis B surface antigen seroreversion after allogeneic hematopoietic SCT. Bone Marrow Transplant (2011) 46(1):125–31. 10.1038/bmt.2010.70 20383209

[64] Nakamoto S Kanda T Nakaseko C Sakaida E Ohwada C Takeuchi M Reactivation of Hepatitis B Virus in Hematopoietic Stem Cell Transplant Recipients in Japan: Efficacy of Nucleos(t)ide Analogues for Prevention and Treatment. Int J Mol ences (2014) 15(11):21455–67. 10.3390/ijms151121455 PMC4264235 25421241

[65] Bae SK Gushima T Saito N Yamanaka I Matsuo Y Yoshida S HBV reactivation after hematopoietic stem cell transplantation and rituximab-containing chemotherapy: a 12-year experience at a single center. Bone Marrow Transplant (2019) 54(4):629–31. 10.1038/s41409-018-0355-6 30287937

[66] Zappulo E Nicolini LA Grazia CD Dominietto A Lamparelli T Gualandi F Efficacy of lamivudine prophylaxis in preventing hepatitis B virus reactivation in patients with resolved infection undergoing allogeneic SCT and receiving rituximab. Infection (2019) 47(1):59–65. 10.1007/s15010-018-1214-5 30232604

[67] Sarin SK Kumar M Lau GK Abbas Z Chan HLY Chen CJ Asian-Pacific clinical practice guidelines on the management of hepatitis B: a 2015 update. Hepatol Int (2016) 10(1):1–98. 10.1007/s12072-015-9675-4 PMC4722087 26563120

[68] Pei SN Ma MC Wang MC Kuo CY Rau KM Su CY Analysis of hepatitis B surface antibody titers in B cell lymphoma patients after rituximab therapy. Ann Hematol (2012) 91(7):1007–12. 10.1007/s00277-012-1405-6 22273839

[69] Cho Y Yu SJ Cho EJ Lee JH Kim TM Heo DS High titers of anti-HBs prevent rituximab-related viral reactivation in resolved hepatitis B patient with non-Hodgkin’s lymphoma. J Med Virol (2016) 88(6):1010–7. 10.1002/jmv.24423 26531242

[70] Murt A Elverdi T Eskazan AE Salihoglu A Ar MC Ongoren S Hepatitis B reactivation in hematopoietic stem cell transplanted patients: 20 years of experience of a single center from a middle endemic country. Ann Hematol (2020) 99(11):2671–7. 10.1007/s00277-020-04206-z 32737632

[71] Bae SK Gushima T Saito N Yamanaka I Shimokawa T Matsuo Y The impact of hepatitis B core antibody levels on HBV reactivation after allogeneic hematopoietic SCT: an 11-year experience at a single center. Bone Marrow Transplant (2016) 51(11):1496–8. 10.1038/bmt.2016.149 27272453

[72] Varma A Biritxinaga L Saliba RM Stich M Jauch SF Afrough A Impact of Hepatitis B Core Antibody Seropositivity on the Outcome of Autologous Hematopoietic Stem Cell Transplantation for Multiple Myeloma. Biol Blood Marrow Transplant (2017) 23(4):581–7. 10.1016/j.bbmt.2017.01.005 PMC5749257 28063964

[73] Lee YC Young KC Su WC Tsao CJ Chen TY Emergence of YMDD mutant hepatitis B virus after allogeneic stem cell transplantation from a HBsAG-positive donor during lamivudine prophylaxis. Haematologica (2004) 89(4):e30–1. 15075101

[74] Seto W-K Chan TSY Hwang YY Mak LY Wong KH Fung J Monitoring and Treatment of Patients Undergoing Immunotherapy With Anti-CD20 Who are Exposed to HBV. Clin Gastroenterol Hepatol (2019) 17(7):1410–2. 10.1016/j.cgh.2018.09.020 30243760

[75] Locasciulli A Alberti A Bandini G Polchi P Arcese W Alessandrino P Allogeneic bone marrow transplantation from HBsAg+ donors: a multicenter study from the Gruppo Italiano Trapianto di Midollo Osseo (GITMO). Blood (1995) 86(8):3236–40. 7579420

[76] Lau G Lie A Kwong Y Lee C Hou J Lau Y A case-controlled study on the use of HbsAg positive donors for allogeneic bone marrow transplantation. Blood (2000) 96(2):452–8. 10.1182/blood.V96.2.452 10887105

[77] Hui C Sun J Au W Lie A Yueng Y Zhang H Occult hepatitis B virus infection in hematopoietic stem cell donors in a hepatitis B virus endemic area. J Hepatol (2005) 42(6):813–9. 10.1016/j.jhep.2005.01.018 15885351

[78] Mallet V Bömmel Fv Doerig C Pischke S Hermine O Locasciulli A Management of viral hepatitis in patients with haematological malignancy and in patients undergoing haemopoietic stem cell transplantation: recommendations of the 5th European Conference on Infections in Leukaemia (ECIL-5). Lancet Infect Dis (2016) 16(5):606–17. 10.1016/S1473-3099(16)00118-3 27599653

[79] Wu Y Shi J Tan Y Zhao Y Yu J Lai X A Novel Strategy for the Prevention of Hepatitis B Virus-Related Hepatitis Following Allogeneic Hematopoietic Stem Cell Transplantation from Hepatitis B Surface Antigen-Positive Donors. Biol Blood Marrow Transplant (2020) 26(9):1719–28. 10.1016/j.bbmt.2020.05.004 32434055

[80] Chiang LT Yao M Ko BS Chen CH Development of immunity against hepatitis B virus after donor lymphocyte infusion in a peripheral blood stem cell transplantation recipient with chronic hepatitis B. Infection (2011) 39(4):363–5. 10.1007/s15010-011-0120-x 21544586

[81] Lindemann M Koldehoff M Fiedler M Schumann A Ottinger HD Heinemann FM Control of hepatitis B virus infection in hematopoietic stem cell recipients after receiving grafts from vaccinated donors. Bone Marrow Transplant (2016) 51(3):428–31. 10.1038/bmt.2015.253 26501767

[82] Brissot E Alsuliman T Beauvais D Bonnin A Mear J-B Souchet L Antiviral prophylaxis for CMV, HSV/VZV and HBV in allogeneic hematopoietic cell transplantation in adult patients: Guidelines from the Francophone Society of Bone Marrow Transplantation and Cellular Therapy (SFGM-TC). Bull Du Cancer (2020) 107(1S):S1–6. 10.1016/j.bulcan.2019.09.002 31627903

[83] Takahata M Hashino S Onozawa M Shigematsu A Sugita J Fujimoto K Hepatitis B virus (HBV) reverse seroconversion (RS) can be prevented even in non-responders to hepatitis B vaccine after allogeneic stem cell transplantation: long-term analysis of intervention in RS with vaccine for patients with previous HBV infection. Transplant Infect Dis (2014) 16(5):797–801. 10.1111/tid.12283 25154638

[84] Chaichotjinda K Anurathapan U Boonsathorn S Chaisavaneeyakorn S Treepongkaruna S Techasaensiri C Immune responses to hepatitis B vaccination after hematopoietic stem cell transplantation in pediatric and young adult patients. Clin Transplant (2020) 34(10):e14024. 10.1111/ctr.14024 32609899

[85] Conrad A Perry M Langlois M-E Labussière-Wallet H Barraco F Ducastelle-Leprêtre S Efficacy and Safety of Revaccination against Tetanus, Diphtheria, Haemophilus influenzae Type b and Hepatitis B Virus in a Prospective Cohort of Adult Recipients of Allogeneic Hematopoietic Stem Cell Transplantation. Biol Blood Marrow Transplant (2020) 26(9):1729–37. 10.1016/j.bbmt.2020.05.006 32428736

[86] Raimondo G Locarnini S Pollicino T Levrero M Zoulim F Lok AS Update of the statements on biology and clinical impact of occult hepatitis b virus infection. J Hepatol (2019) 71(2):397–408. 10.1016/j.jhep.2019.03.034 31004683

[87] Hammond SP Borchelt AM Ukomadu C Ho VT Baden LR Marty FM Hepatitis B virus reactivation following allogeneic hematopoietic stem cell transplantation. Biol Blood Marrow Transplant (2009) 15(9):1049–59. 10.1016/j.bbmt.2009.05.001 19660717

[88] Wei J Zhu X Mao X Huang L Zhou J Severe early hepatitis B reactivation in a patient receiving anti-CD19 and anti-CD22 CAR T cells for the treatment of diffuse large B-cell lymphoma. J Immuno Ther Cancer (2019) 7(1):315. 10.1186/s40425-019-0790-y PMC6868854 31753002

[89] Strati P Nastoupil LJ Fayad LE Samaniego F Neelapu SS Safety of CAR T-Cell Therapy in Patients with B-Cell Lymphoma and Chronic Hepatitis B or C Virus Infection. Blood (2019) 133(26):2800–2. 10.1182/blood.2019000888 PMC7265784 31101626

[90] Yang C Xie M Zhang K Liu H Liang A Young KH Risk of HBV reactivation post CD19-CAR-T cell therapy in DLBCL patients with concomitant chronic HBV infection. Leukemia (2020) 34:3055–9. 10.1038/s41375-020-0913-y 32533094

[91] Rui C Cuicui L Qing L Yanyu J Nan M Zhenxing Y Humanized anti-CD19 CAR-T cell therapy is safe and effective in lymphoma and leukemia patients with chronic and resolved HBV infection. Hematol Oncol (2020). 10.1002/hon.2807 PMC7983916 32949412

[92] Hammond SP Chen K Pandit A Davids MS Issa NC Marty FM Risk of hepatitis B virus reactivation in patients treated with ibrutinib. Blood (2018) 131(17):1987–9. 10.1182/blood-2018-01-826495 29490923

[93] Malek AE Nieto Y Szvalb AD Siddiqui S Torres HA Hepatitis B Virus-associated Liver Failure in a Patient With B-cell Non-Hodgkin Lymphoma After Anti-cancer Therapy Including Ibrutinib. Clin Lymphoma Myeloma Leukemia (2020) 20(3):e124–e7. 10.1016/j.clml.2019.12.006 PMC8262650 31932250

[94] Innocenti I Morelli F Autore F Corbingi A Pasquale R Sorà F HBV reactivation in CLL patients with occult HBV infection treated with ibrutinib without viral prophylaxis. Leukemia Lymphoma (2019) 60(5):1340–2. 10.1080/10428194.2018.1523401 30730231

